# Landscape, Environmental and Social Predictors of Hantavirus Risk in São Paulo, Brazil

**DOI:** 10.1371/journal.pone.0163459

**Published:** 2016-10-25

**Authors:** Paula Ribeiro Prist, Maria Uriarte, Leandro Reverberi Tambosi, Amanda Prado, Renata Pardini, Paulo Sérgio D´Andrea, Jean Paul Metzger

**Affiliations:** 1 Department of Ecology, Bioscience Institute, University of São Paulo, São Paulo, SP, Brazil; 2 Department of Ecology, Evolution & Environmental Biology, Columbia University, New York City, NY, United States of America; 3 Department of Zoology, Bioscience Institute, University of São Paulo, São Paulo, SP, Brazil; 4 Department of Tropical Medicine, Oswaldo Cruz Institute, FIOCRUZ, Rio de Janeiro, RJ, Brazil; University of Texas Medical Branch at Galveston, UNITED STATES

## Abstract

Hantavirus Pulmonary Syndrome (HPS) is a disease caused by Hantavirus, which are negative-sense RNA viruses in the family *Bunyaviridae* that are highly virulent to humans. Numerous factors modify risk of Hantavirus transmission and consequent HPS risk. Human-driven landscape change can foster transmission risk by increasing numbers of habitat generalist rodent species that serve as the principal reservoir host. Climate can also affect rodent population dynamics and Hantavirus survival, and a number of social factors can influence probability of HPS transmission to humans. Evaluating contributions of these factors to HPS risk may enable predictions of future outbreaks, and is critical to development of effective public health strategies. Here we rely on a Bayesian model to quantify associations between annual HPS incidence across the state of São Paulo, Brazil (1993–2012) and climate variables (annual precipitation, annual mean temperature), landscape structure metrics (proportion of native habitat cover, number of forest fragments, proportion of area planted with sugarcane), and social factors (number of men older than 14 years and Human Development Index). We built separate models for the main two biomes of the state (cerrado and Atlantic forest). In both biomes Hantavirus risk increased with proportion of land cultivated for sugarcane and HDI, but proportion of forest cover, annual mean temperature, and population at risk also showed positive relationships in the Atlantic forest. Our analysis provides the first evidence that social, landscape, and climate factors are associated with HPS incidence in the Neotropics. Our risk map can be used to support the adoption of preventive measures and optimize the allocation of resources to avoid disease propagation, especially in municipalities that show medium to high HPS risk (> 5% of risk), and aimed at sugarcane workers, minimizing the risk of future HPS outbreaks.

## Introduction

Multiple lines of evidence suggest that ecological and anthropogenic factors play important roles in elevating incidence of diseases around the world [1]. Landscape composition (e.g., relative abundance of landscape units) and configuration (e.g., spatial arrangement of landscape units) may affect disease incidence by altering interactions, abundance, and movements of hosts, vectors, and people, although the effects of these landscape variables on disease dynamics are understood only for a handful of well-studied cases [2]. For instance, in the Amazon Basin and East Africa, deforestation increases standing water and sunlight, and enhances breeding success of some mosquito species, which can increase risk of malaria transmission [3]. Habitat fragmentation and decreasing habitat patch size increase the risk of Lyme disease transmission in North America [4; 5] and Hantavirus Cardiopulmonary Syndrome (HPS) transmission risk in Panamá [6].

HPS ranks among the major emerging diseases of the last century, and is expected to remain a public health threat into the future [7]. It was first recognized in May 1993 in the Four Corners region of the US [8], and a few months later, in the city of Juquitiba, in the state of São Paulo, Brazil [9]. Rodents in the family Cricetidae are the primary hosts of HPS in Brazil [10; 11], a virus (family *Bunyaviridae*) that causes two syndromes in humans: HPS in the Americas, and hemorrhagic fever with renal syndrome (HFRS) in Eurasia and Africa [11]. Transmission to humans occurs via inhalation of aerosolized virus particles derived from the urine, saliva, and feces of infected rodents [12; 13]. HPS is associated with high lethality rates (35% in the US; 41% in Brazil; 38% in Canada) [14; 9; 15].

Currently, most studies support the hypothesis that forest loss, forest fragmentation, and anthropogenic landscape change, as consequences of natural habitats conversion to agricultural areas increases prevalence of Hantavirus in reservoir species [16; 6; 17; 18]. This effect occurs because these species are generally habitat generalists [19; 20] that can tolerate and adapt to ecological changes [21], being favored in disturbed environments [6; 17] and becoming abundant in altered landscapes [19; 6; 22; 23]. In addition, greater population densities of these reservoir species increase intraspecific encounters and consequent Hantavirus transmission [20; 16].

Climate can also influence host rodent population abundance and Hantavirus transmission dynamics. Several studies in North America have uncovered positive associations between precipitation, population size of rodent hosts, and Hantavirus prevalence [24; 10; 25]. High precipitation increases vegetation growth, boosting rodent densities and enhancing probability of human-rodent encounters and consequent Hantavirus transmission [26; 27]. Temperature may also affect reproduction and survival rates of small rodents, as well as the time that the virus remains infectious in the environment [28]; these effects influence transmission risk, although their direction is not entirely clear [29].

Additionally, several socio-economic variables can also influence disease transmission. HPS epidemiology is complex, involves many factors, and the distribution and abundance of the reservoir species does not necessarily imply transmission of the disease. Agricultural practices (such as mechanization, use of personal protective equipment and adequate infrastructure to handle and stock production), public sanitation, types of preventive measures used, education, behavior, and economic conditions also influence HPS transmission [30].

The relationship between landscape, climate, and social factors associated with Hantavirus transmission remains unexamined, particularly in Latin America. Evaluating the relative contributions of these factors to Hantavirus transmission can enable predictions of future outbreaks, and can be critical to design effective surveillance, control, and mitigation programs. Here, we rely on a Bayesian model to fill this research gap for the state of São Paulo, Brazil: we quantify associations between HPS incidence and the size of risk populations (e.g., number of rural men older than 14 years) and potential drivers including landscape structure (e.g., percentage of landscape units and fragmentation of native habitats), climate (e.g. temperature, precipitation), and social factors (Human Development Index, HDI dismembered, poverty and Gini Index). We make the following predictions:

1. HPS incidence will be greater in municipalities with a lower proportion of native habitat cover, a large proportion of agriculture and habitat edge areas, and with a large number of fragments, because HPS reservoir species are habitat generalist which increase in abundance in edge habitats and in agricultural landscapes;
2. HPS incidence will be greater at higher precipitation, once it affects rodent population dynamics, increasing their abundance;
3. HPS incidence will be greater in municipalities with lower human development index (HDI), and with a large number of population at risk, since economic and social conditions can also affect HPS transmission, and the bigger the number of people in contact with infected rodents the greater is the chance of HPS transmission. We then use the results to identify high-risk areas for HPS incidence across the state of São Paulo.

## Materials and Methods

### Study Area

**Fig 1:**
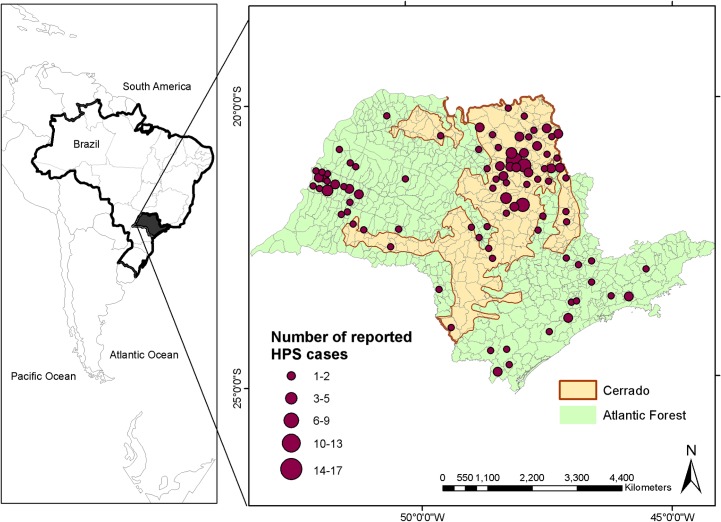
Hantavirus incidence between 1993 and 2012 across the 645 municipalities of the state of Sao Paulo, and cerrado (in orange) and Atlantic forest (in green) delimitation in the state.

We focused analyses in São Paulo, Brazil, including both the cerrado and Atlantic forest biomes (Fig 1), in southeastern Brazil, with an area of ~248,210 km^2^, and a population of ~42 million (21.5% of Brazil’s population); [31]. At present, only 13% of the state of São Paulo is still covered by remnants of its original biomes (cerrado and Atlantic forest), with the remaining area being covered by agriculture, especially a mixture of sugarcane plantations [32], pasture [33], and urban landscapes. Cerrado covered originally 33% of the state [34], but now almost 81% of its area is converted to anthropic uses [34]. It comprises a mosaic of vegetation types ranging from savanna with sparse shrubs and small trees to almost-closed woodland [35]. The region sees rainy summers and dry winters, with annual precipitation of 1390 mm [35]. The Atlantic forest originally covered ~69% of the state [36]. Only 13.9% of the original vegetation remains, and is now highly fragmented [36]. These areas are characterized by hot and rainy summer, without a defined dry season [37]; annual precipitation ranges 1000–2200 mm [38].

### Disease and social data

HPS incidence data are collected at the municipality level, so we treated the 645 municipalities within São Paulo state as our sampling units. The number of reported HPS cases in each municipality per year from 1993 to 2012 was extracted from the website of the Center for Epidemiological Surveillance of the State of São Paulo (CVE-SP) (1993–2012), and the Health Portal SUS (http://portal.saude.gov.br/portal/saude/profissional/area.cfm?id_area=1558). The CVE-SP compiles this information, once the cases of Hantavirus infection are of mandatory notification to the local health authorities in Brazil. The data is provided by every hospital in the state, once the patients' living address is a confidential information. Therefore CVE considers all HPS cases confirmed by laboratory analysis (antibody positive) that were recorded in any hospital presented in each municipality of São Paulo state. Since the majority of the municipalities had 0 (98.39%) or 1 case per year (1.61%), with the maximum of 4 cases per municipality per year, we transformed the data to binary, reflecting presence *versus* absence of HPS. Then, we modeled the probability of incidence of HPS in each municipality based on the reported cases from 1993 to 2012 and covariates reflecting social, climatic and landscape condition described on the next paragraphs. In that way, the probability of HPS risk was defined as the probability of an HPS infection to occur in a municipality.

Epidemiologic data indicate that more than 70% of HPS-infected people was working or living in agricultural areas, and ~93% were men over the age of 20 [39; 40; 41]. Because the available data are relatively coarse with respect to age distribution, we used the number of rural men older than 14 years in each municipality as the population at risk for HPS. This information was extracted from the National Institute of Geography and Statistics (IBGE) website (www.ibge.gov.br), and was available only for 1996 and 2006. Since we wanted to model the incidence of HPS from 1993 to 2012, we thus used the 1996 data as covariates to predict disease incidence for 1993–2001, and 2006 data to predict incidence for 2002–2012.

Since socio-economic development can influence HPS transmission, Human Development Index (HDI), HDI elements including life expectancy, income, and education, Gini index and poverty were tested for association with HPS incidence across São Paulo. The Human Development Index (HDI) is a summary measure of average achievement in key dimensions of human development and socioeconomic status of the populations, and includes elements of life expectancy, income (GDP/capita), and education. HDI is thus a measure of human development and poverty, and can be used as a proxy of the socio-economic factors (education, poverty and health) that influence HPS risk [42]. HDI data at the municipality level were extracted from IBGE (www.ibge.gov.br) website, with data available for 1991, 2000, and 2010. We used HDI data from 1991 as covariates to predict incidence for 1993–1998, data from 2000 to predict incidence for 1999–2005, and data from 2010 to predict incidence for 2006–2012. HDI elements (e.g., life expectancy, income, and education) were extracted from United Nations Development Programme (UNDP) (http://www.pnud.org.br/arquivos/ranking-idhm-2010.pdf), with data available only for 2010. Gini index is used to measure inequality, and together with poverty, was extracted from IBGE (www.ibge.gov.br) being available only for 2003. Therefore, we used HDI elements data from 2010 and Gini and poverty data from 2003 as covariates to predict incidence for the entire period (1993–2012).

### Landscape composition and configuration metrics

We used the São Paulo state forest inventory map (http://www.iflorestal.sp.gov.br) for the years 2000 and 2010 to calculate landscape composition and configuration metrics for each municipality. This native vegetation inventory covers two dates in our study period (2000 and 2010), and was generated at a 1:50.000 scale, with a minimum mapped area of 2.5 ha, being able to identify small fragments, which are very common in the São Paulo state. Additionally, using the IF inventory made possible to use information with same spatial resolution and mapping method for both cerrado and Atlantic Forest biome in the state. Native vegetation cover aggregated both Atlantic Forest and cerrado remnants, and we considered each municipality as individual landscapes for analysis. Landscape composition was measured considering the relative abundance of each landscape unit (percentage of native vegetation cover—forest and cerrado; and sugarcane, pasture and corn), while landscape configuration refers to the degree of fragmentation of native vegetation cover types (forest and cerrado), measured by the number of habitat fragments (e.g., number of forest or cerrado patches in a landscape) and density of habitat edge (e.g., total length of habitat/non-habitat edge per area of landscape). A municipality was considered as part of the Atlantic Forest or cerrado biome depending on the percentage of its area that overlapped the distribution of these biomes (see Statistical analyses).

All landscape analyses were done in ArcGis 10.0 and Fragstats 4.2. We used metrics extracted from the 2000 map as covariates to model incidence for 1993–2001, and metrics extracted from the 2010 map as covariates for period 2002–2012.

The main agricultural land uses in São Paulo—sugarcane [43], pasture [44], and corn [16]—result in habitats favorable to generalist rodent species achieving high abundances [45]. Some of these land uses have a relatively high temporal heterogeneity (e.g., massive biomass production from planting to harvest in a few months or years, providing considerable amounts of high-energy food) [43; 45; 46; 47]. Small rodents take advantage of this tremendous food supply, increasing their abundances [47], which may influence Hantavirus incidence [48]. Therefore, we also used data for these agricultural land uses, to test for associations with Hantavirus incidence. We obtained annual data from the Agricultural Census of the Institute of Agricultural Economics (www.iea.sp.gov.br) to use the proportion of sugarcane, pasture, and corn in each municipality as covariates to model annual disease incidence for 1993–2012.

### Climatic variables

Meteorological data used were obtained from the International Research Institute for Climate and Society (IRI) Data Library (http://iridl.ldeo.columbia.edu/index.html). Gridded land surface temperature data were obtained from National Centers for Environmental Prediction (NOAA NCEP) from combined GHCN and CAMS station data at 0.5° spatial resolution, and extracted for each municipality (as average value across the municipality). The monthly data [49] were used to calculate annual mean, minimum, and maximum, and seasonal mean, minimum, and maximum temperature values, for each municipality over 1993–2012.

Precipitation data were obtained from the University of California Santa Barbara from the Climate Hazards Group Infrared Precipitation with Stations (CHIRPS) data set, with a spatial resolution of 0.05°, and extracted for each municipality (as average value across the municipality). The 10-day averages data [50]; were used annual mean, minimum, maximum, and total, and seasonal mean, minimum, and maximum, precipitation for each municipality for 1993–2012.

### Statistical analysis

Hantavirus shows high host specificity. Therefore, as expected, for each Brazilian region there are different reservoir species hosting distinct virus strains [7]. Although some geographic overlap occurs [51], Araraquara virus (ARAV) is the dominant pathogenic Hantavirus in cerrado, and is commonly associated with HPS cases there [51; 52], whereas Juquitiba (JUQV) is the dominant pathogenic Hantavirus in Atlantic forest [51]. Given the geographic distribution of the two viruses, and the assumption that *Oligoryzomys nigripes* is the chief reservoir for human HPS cases in Atlantic forest [53] and *Necromys lasiurus* is the reservoir in cerrado, Hantavirus transmission risk was modeled separately in the two biomes. Municipalities were considered as cerrado or Atlantic forest if >50% of their surface area fell inside one or the other biome. Biome distribution was obtained from IBGE (www.ibge.gov.br).

To reduce numbers of predictor variables we fitted and compared generalized linear mixed models (see further detail on methods and results of exploratory analyses in S1 to S3 Tables). We then fitted a Bayesian model containing only 7 predictor variables as fixed covariates: proportion of sugarcane, proportion of native vegetation cover, number of native vegetation patches, HDI, mean annual temperature (°C), total annual precipitation (mm), and rural male population >14 years old (S4 Table). All variables included in the model had correlations <0.4 relative to other variables. Non-linear correlations between variables were assessed visually; when necessary, a quadratic form was fit to covariates and compared with linear relationships using the Deviation Information Criterion (Bayesian method for model comparison; [54]). In every case, the linear form provided a better fit to the data. Percent of native vegetation cover and annual precipitation were log-transformed prior to analysis.

Municipality was included as a random effect to account for differences among administrative units not captured in the fixed covariates. To facilitate interpretation, all estimated parameters were standardized, centered on their means, and divided by two standard deviations [55].

All priors were assigned as uninformative distributions. We used the *rjags* package in R, and examined model convergence and performance via Gelman-Rubin diagnostics. Parameters were considered significant if the 95% quantiles of their distribution did not overlap 0. We also calculated Bayesian *p*-values to examine discrepancies between means of simulated and real data (e.g., values close to 0.5 represent a good model; [56], and *R*^2^ to examine the square of the correlation between true and predicted outcomes [57]. As HPS can be considered as a rare event in the state of São Paulo (1% of success) we did not validate our model by test-training procedure (e.g., removing a random part of data points to fit the model to the remaining data), as it was necessary to have all data available for calibrating the model. Therefore, we are using Bayesian *p*-values and *R*^2^ as measures of validation.

We tested HPS incidence and model residuals for both models, constructed for Atlantic forest and cerrado biomes, for spatial autocorrelation, by calculating Moran’s *I*. For this analysis we used the spatial contiguity matrix based on the Queen´s case neighborhood relation and treat each year, from 1993 to 2012, separately. This test is commonly used and accepted as a fair evaluation of spatial autocorrelation and dependence [58], especially in disease studies [59, 60, 61]. For both models and for HPS incidence, Moran´s *I* results showed no spatial autocorrelation (see S5 and S6 Tables) for the majority of years, justifying our use of a non-spatial model.

Mapping is a primary goal in spatial epidemiology [62], as it allows immediate visualization of the extent and magnitude of public health threats [63]. We used model results to generate a map of Hantavirus risk areas for the state of São Paulo. Risk was defined as the probability of an HPS infection to occur in a municipality. The mean and coefficient of variation of simulated results among years were summarized for each municipality, and imported into ArcGIS 10.0 for visualization.

A t-test or an one-way analysis of variance (ANOVA), followed by Tukey’s Multiple Comparison Test, were performed to check whether significant differences existed in the final predictor variables between available years.

## Results

**Fig 2:**
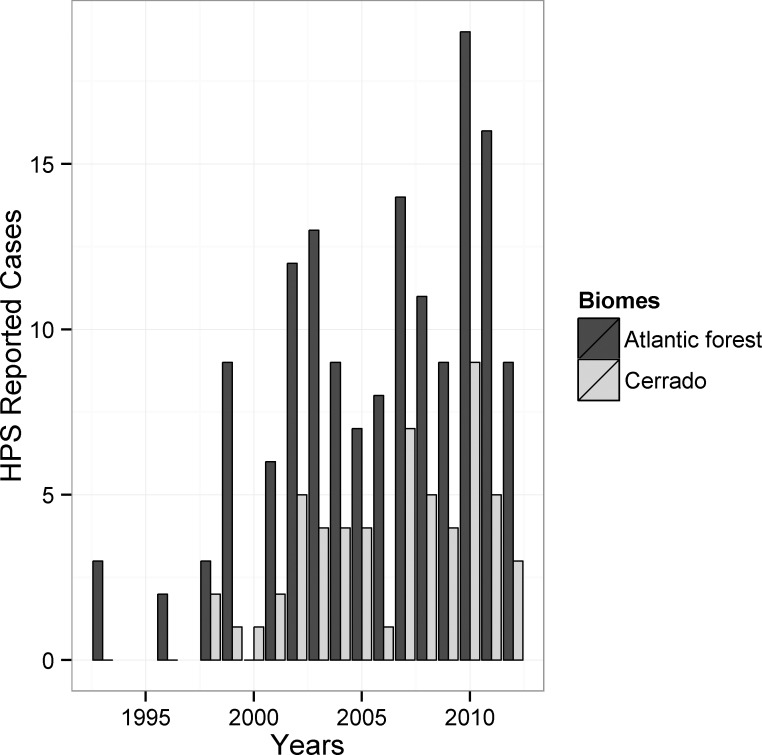
Reported HPS cases between 1993 and 2012 in the cerrado and Atlantic forest regions of the state of São Paulo.

During 1993–2012, 207 HPS cases were reported for the state, with increasing numbers in the last 10 years (Fig 2). Of the total, 57 cases were reported from the cerrado region (161 municipalities; 0.35 cases per municipality) whereas 150 (484 municipalities; 0.30 cases per municipality) in the Atlantic forest region. The largest number of HPS cases are concentrated in the northeastern region, followed by the western region, although there are cases statewide (Fig 1). Increases in HPS incidence were particularly marked in the 2000s, raising was 250% if compared to the number of cases in the 1990s. Only in 2005 we can observe a decrease in the number of reported cases especially for cerrado region, but after this period it augment further, reaching a peak of 28 cases only in 2010 (Fig 2).

The majority of the factors considered in our model exhibited significant trends over the study period. Proportion of native vegetation cover increased between 2000 and 2010 for both cerrado (8.10 to 11.38%) and Atlantic forest (12.16 to 16.20%) municipalities, despite concomittant increases in proportion of sugarcane cultivated in these regions over the same period (Table 1). The average number of native vegetation patches also increased in both cerrado (208 to 557) and Atlantic forest (149 to 413) regions (Table 1). HDI and the size of the population at risk (e.g., males > 14 yrs old) also increased over the years for which data were available in both regions (Table 2); annual mean temperature and annual precipitation did not show any significant trend (S7 Table).

**Table 1:** Average and range values of percent of sugarcane cultivated, percent of native vegetation cover, and number of patches for the municipalities of cerrado and Atlantic forest regions, for the years 2000 and 2010.

Cerrado			
Year	Native vegetation cover	Sugarcane	Number of patches
2000	8.10 (0.65–79.24)***	15.44 (0–86.39)***	208.39 (11–1869)***
2010	11.38 (1.9–82.34)	27.60 (0–81.72)	557.12 (31–3624)
Atlantic forest			
2000	12.16 (0.17–89.3)***	11.57 (0–91.6)***	149.84 (2–925)***
2010	16.20 (0.06–91.8)	22.95 (0–97.85)	413.36 (1–2611)

*** represents significant difference among years.

**Table 2:** Average and range values of HDI (Human Development Index) and Population at risk (rural men older than fourteen years) for the municipalities of cerrado and Atlantic forest regions, for the years that the data is available.

	Human Development Index			Population at Risk	
Year	cerrado	Atlantic forest	Year	Cerrado	Atlantic forest
1991	0.50 (0.31–0.64)***	0.49 (0.23–0.69)***	1996	1209 (4–4610)	993 (2–7473)
2000	0.65 (0.5–0.74)***	0.64 (0.46–0.82)***	2006	1327 (10–7085)	1052 (1–7549)
2010	0.74 (0.65–0.82)	0.73 (0.63–0.86)			

*** represents significant difference among years.

Overall, our statistical models fit well to the data (cerrado: *R*^2^ = 0.19; Bayesian *p*-value = 0.49; Atlantic forest: *R*^2^ = 0.23; Bayesian *p*-value = 0.50), and showed significant effects of landscape, climate, and social variables on Hantavirus infection risk, as is described below in detail.

**Fig 3:**
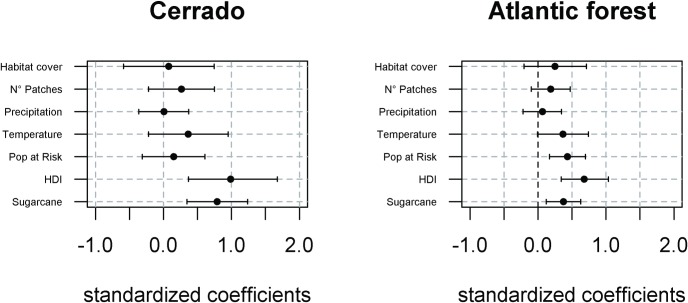
Parameter estimates with mean (black dot) and credible intervals (2.5–97.5%) of predictors of HPS risk for cerrado and Atlantic forest region. Habitat cover = percentage of native vegetation cover; N° Patches = number of native vegetation fragments; Precipitation = total annual precipitation; Temperature = mean annual temperature; Pop at Risk = population at risk, i.e. rural men aged over 14 years; HDI = Human Development Index; Sugarcane = percentage of municipality occupied by sugar cane plantations.

In cerrado, probability of Hantavirus infection risk was significant and positively related to HDI and to proportion of municipality occupied by sugarcane plantation (Fig 3). Number of patches and annual mean temperature also showed a positive relationships, but they were not significant, with greater risk of HPS infection in more fragmented habitats and in years with higher annual mean temperatures (Fig 3).

For Atlantic forest, population at risk, HDI, and proportion of sugarcane were all significantly positively associated with Hantavirus infection risk (Fig 3). Annual mean temperature and number of patches had marginally significant positive relationships to Hantavirus infection, with a higher chance of Hantavirus infection in municipalities with higher temperatures and more fragmented forests.

**Fig 4:**
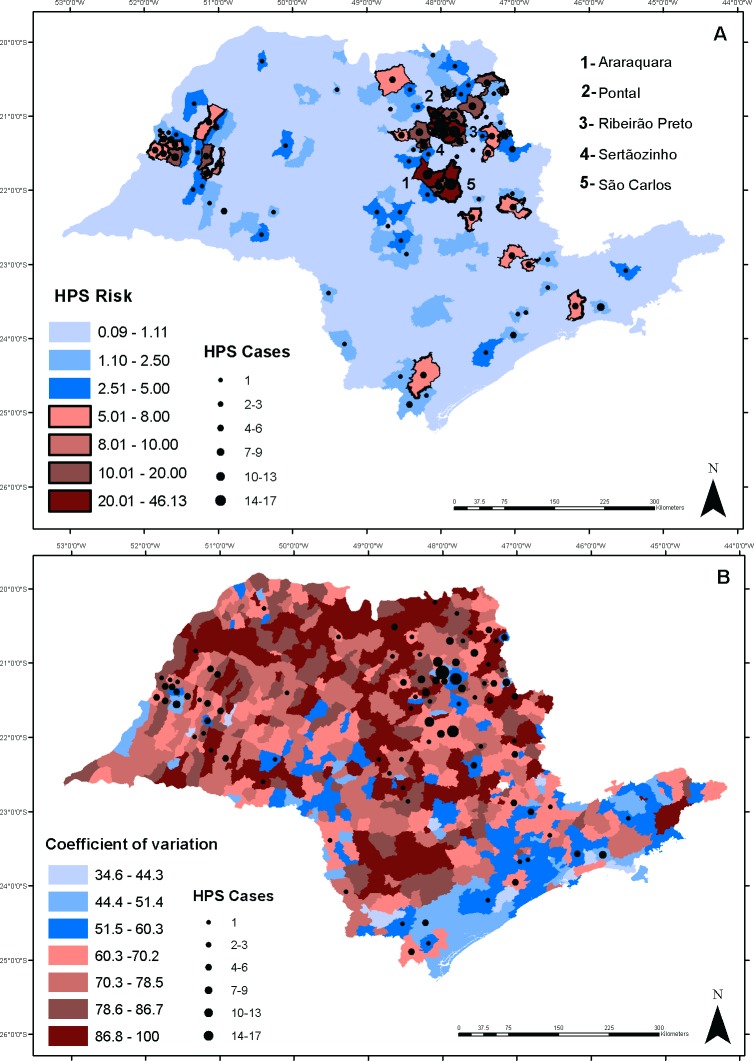
**Map of Hantavirus infection probability (%) (A) mean (B) and coefficient of variation (B) among years across the state of São Paulo.** Black dots depict number of reported cases between 1993 and 2012. Municipalities with no symbol means no reported cases of HPS. Black outlines indicate municipalities with medium to high risk (> 5%) of Hantavirus infection and where preventive effort should be allocated.

Using results from the statistical models, we mapped HPS risk across the state. Overall, 6% of the state was classified as medium (5–10%) or high (> 10%) risk category for HPS infection, and 94% was indicated as low risk (<5%) category (Fig 4A). All municipalities with a medium to high risk of Hantavirus infection are shown with black outlines in the risk map and represent municipalities where preventive measures should be allocated.

Not surprisingly, municipalities with highest mean risk were those that already had many HPS cases (Fig 4A). Municipalities in the northeastern region have particularly high mean risk (up to 46%), followed by some municipalities of the east, close to the Serra do Mar (in Atlantic forest region) and in the west of the state, with a mean risk up to 21%. The municipalities of São Carlos, Ribeirão Preto, Pontal, Sertãozinho and Araraquara, located in the northeast of the state (Fig 4A), are the ones that present a very large mean risk for Hantavirus infection (> 20%). The coefficient of variation of Hantavirus infection risk across years (Fig 4B) is smaller in the eastern part of the state, where habitat cover is relatively high (S1A Fig). Based on our model, a large number of municipalities that have not registered HPS infection have HPS risk of up to 2.5% (Fig 4A). At the same time, some municipalities that had HPS cases have a small risk of Hantavirus infection (8% of the state). Maps with the minimum and maximum risk are shown in S2 Fig.

## Discussion

This study is the first to link landscape structure, climate, and social variables to Hantavirus infection risk in the Neotropics. Our model identified >6% of the state of São Paulo as presenting medium-to-high risk of Hantavirus transmission (39 municipalities). This disease that has so far killed at least 99 people (47.8% lethality rate) in the state, and 637 people in Brazil, since 1993 [9]. A key finding of our study is that the extent of sugarcane plantations was the most important predictor of Hantavirus infection, and this relationship held in both the cerrado and the Atlantic forest. Increases in HPS incidence were noted in the 2000s, when high oil prices led to substantial expansion of sugarcane cultivation in the state [64].

Sugarcane plantations support greater abundances of rodents than other ecosystems, whether natural or agricultural [16; 43; 65]. Brazil is the largest producer (~ 490 x 10^6^ tons/year in 2011–2012) and exporter of sugarcane in the world [66], with most of this production (~76%) in São Paulo [67]. Yields in these plantations can reach 120 tons/year/ha [68], serving as a readily available source of food and cover for rodents [46]. Moreover, in recent years sugarcane cultivation has become increasingly mechanized, as a result of legislation limiting burning prior to harvest (Law n°. 11.241, 19 September, 2002). These shifts in predominant harvest mode may have reduced rodent mortality (large numbers previously were killed by burning), increasing population sizes, and consequently augmenting Hantavirus infection risk [41; 65; 47]. Sugarcane cultivation is projected to increase still further in coming decades [69], with implications for HPS risk across São Paulo and neighboring states in southern and central Brazil.

Surprisingly, high HDI was associated with increased HPS incidences in both regions. This result was unexpected because a number of studies have found no relationship between Hantavirus infection and socio-economic status [40; 41]; others studies have found poor sanitary and living conditions to be positively associated with incidence [13; 70]. For São Paulo, however, the positive association between HDI and HPS risk may reflect better socioeconomic conditions in municipalities where sugarcane is dominant economically: sugarcane municipalities have, on average, stronger social welfare indicators [71; 72], with this sector contributing to the concentration of income [71], even outperforming the greater São Paulo Metropolitan Region [71].

HPS studies elsewhere have found that most individuals affected by HPS participate in agricultural or forestry activities [30; 40; 41; 42; 72]. This connection exists because HPS transmission requires contact between humans and aerosolized excreta of infected rodents, which is most likely in this demographic group. This pattern was clear for the Atlantic forest, which showed a positive relationship between population at risk (number of males older than 14 years old) and HPS incidence; however, this relationship did not hold for the cerrado. Another study in the cerrado [73] also failed to uncover an association between Hantavirus infection and the size of rural populations. One possible reason for absence of such association may be that cerrado municipalities have a longer history of sugarcane cultivation and more land proportionally cultivated for sugarcane than Atlantic forest municipalities (S1B Fig); this crop also brings large numbers of temporary workers from other states who do not end up in the official population statistics [74], which may have reduced the strength of any association between the size of the population at risk and HPS incidence. Another possibility is that some Atlantic forest municipalities have taken longer to achieve large-scale mechanization (e.g., in 2007, regions such as Pindamonhangaba and Guaratinguetá still had 0% mechanization; [75]), leading to greater transmission probabilities for workers in this biome. Workers in unmechanized plantations may have greater probability of contact with rodent excreta, as unmechanized plantations use workers in all steps of the production process, whereas in mechanized systems they work only in some steps of the process [75].

In Atlantic forest, our model showed marginal positive associations of fragmentation with Hantavirus incidence, supporting studies elsewhere [6; 17; 76], whereas in cerrado this association was not significant. The same result was found for proportion of habitat cover. In tropical regions HPS risk is expected to be higher in areas with a small proportion of habitat cover [6; 17], which contrasts with our results for Atlantic forest. For cerrado, the proportion of habitat cover had no association with HPS risk. We hypothesize that this may reflect the fact that most of municipalities within Atlantic forest have a small amount of habitat cover (~8.6%), and are composed of second-growth forests in early to medium stages of succession [77]. Both hantavirus reservoir rodent species in São Paulo (*N*. *lasiurus* and *O*. *nigripes*) are habitat generalists [43; 78]. While *O*. *nigripes* is known to survive in small and isolated forest remnants [23; 79], and prefers early successional stages inside the forest [78; 79; 80], becoming more abundant in these landscapes, *N*. *lasiurus* seems to prefer less dense and open areas [43; 81], occurring in disturbed and open habitats [78].

High annual mean temperature was marginally associated with greater HPS risk in the Atlantic forest. For cerrado, this association was not significant. This result supports findings elsewhere [26; 82; 83; 84]. Temperature can affect vegetation growth [29] and the survival rate of rodents [85], with mild temperatures (10–25°C) being most favorable for rodent breeding [86]. Additionally, reservoir rodents normally exhibit a peak in hantavirus infection during warmer months [87; 88; 89; 90], probably because high temperature leads to greater aerosolization of the virus and higher rates of inhalation by both humans and rodents [12; 13]. At present there are no data available on the effects of temperature on HPS virus survival, but laboratory experiments found that Puumala viruses (aetiological agent of Hantavirus infection in Western Europe) can remain infectious for longer (i.e.12 to 15 days) at room temperature (23°C; 73°F) [91], losing their viability if kept at 37°C [91]. The average temperature for São Paulo municipalities, from 1993 to 2012 was 22.9° C, with maximum temperature of 27° C, while the highest HPS infection risks were found between 22.5°C and 25°C, intermediate conditions for São Paulo state. This temperature range would be ideal for virus survival in nature, if JUQV and ARAV virus conditions are the same as for the Puumala virus. Studies conducted over shorter spatial scales than the work presented here would be necessary to link climatic factors to HPS incidence.

There was no effect of precipitation on Hantavirus risk, even in the cerrado, which has a marked dry season [92]. The association between precipitation and Hantavirus infection is still controversial, with no association in some studies [83; 93], negative effects in others [85], while in others increased rainfall in fall to spring have resulted in higher HPS transmission [26; 27], and in rodent outbreaks [24]. These studies, however, were performed in arid and semi-arid regions (85–100 mm precipitation per year) [24; 94], which show an increase in both rodent richness and abundance in response to high precipitation [24; 94]. Since our study was performed in a tropical region where annual mean precipitation is considerably high, the effects of this climatic variable on rodent resources and population dynamics may not be important.

According to our risk map, a large number of municipalities that had no reported cases nevertheless have some HPS risk (0.9–4.9%). Although this risk may seem small, the combination of lack of effective prevention and treatment options (with high lethality makes HPS a serious public health risk. Additionally, as rare events (~1% of events) and undiagnosed asymptomatic infections that are typically not reported to official statistics [70], these numbers may be underestimated. This situation is of special concern in view of the high variation of infection risk in several municipalities that had no HPS infections reported. Due to its high lethality, an infection risk up to 5% should be considered as a medium risk, and municipalities with risk higher than this value or with very high coefficient of variation should be included in preventive measures programs. Northeastern municipalities showed the highest risk across the state (up to 46%), probably because sugarcane cultivation is intense in that region: it is essential to implement awareness campaigns and reinforce diagnostic protocols to detect HPS infections in these municipalities, especially in sugarcane properties. Simple, low cost measures such as use of personal protective equipment in any environment where wild rodent excreta is frequently present, rodent control and proper clean-up of their excrement in human dwellings, seal homes against the entry of rodents, and apply other rodent-proofing techniques, may go a long way toward reducing HPS incidence and human mortality.

## Supporting Information

S1 Table Exploratory analysis results made with generalized linear mixed models. (DOCX)

S2 Table Exploratory analysis results made with generalized linear mixed models and rodent abundance data. (DOCX)

S3 Table All set of candidate models analyzed in the generalized linear mixed models. (DOCX)

S4 Table Predictor variables included in the model. (DOCX)

S5 Table Moran´s I test applied to the residuals of the Bernoulli models for Cerrado and Atlantic forest regions. (DOCX)

S6 Table Moran´s I test applied to the number of HPS cases for cerrado and Atlantic forest regions. (DOCX)

S7 Table Average and range values of annual mean temperature and total precipitation for the municipalities of Cerrado and Atlantic Forest regions, from 1993 to 2012. (DOCX)

S1 Fig Amount of native vegetation (A) and sugar cane plantation (B) in the state of São Paulo.
(DOCX)

S2 Fig Spatial representation of the minimum (A) and maximum (B) probability of Hantavirus infection risk for São Paulo State.
(DOCX)
