# Supplementary material for: Landscape, Environmental and Social Predictors of Hantavirus Risk in São Paulo, Brazil
Source: PLoS One. 2016 Oct 25;11(10):e0163459. doi: 10.1371/journal.pone.0163459 (PMC5079598; doi:10.1371/journal.pone.0163459)
Supplement: S1 Table — (DOCX) [file pone.0163459.s001.docx]

Landscape, environmental and social predictors of Hantavirus risk in São Paulo, Brazil

Paula Ribeiro Prist^1*^, Maria Uriarte^2^, Leandro Reverberi Tambosi^1,2^, Amanda Prado^1^, Renata Pardini^3^, Paulo Sérgio D´Andrea^4^, Jean Paul Metzger^1^

**Supplementary** **Material**

**Exploratory analysis**

We fitted generalized linear mixed models to reduce the number of predictor variables among landscape, climate and social factors to be included in the final Bayesian model. We split the data into cerrado and Atlantic Forest biomes and used "lme4" package in R program version 3.03, with a Binomial family error distribution. Precipitation and temperature variables were selected through the comparison of 21 simple models, for each biome, each containing one of the 21 climatic variables as fixed factor, municipality and year as random effects, and the presence of HPS cases as the response variable. Only the variables included in significant models were selected to enter in the model selection with landscape, social and agricultural variables (e.g., annual mean temperature, mean temperature of winter, annual total precipitation and annual maximum precipitation).

After that we performed a maximum likelihood model selection procedure, considering the second-order Akaike´s information criterion (AIC) (Burnham and Anderson 2002) to compare a set of 216 candidate models, for each biome, combining climate, social, landscape and agricultural variables, including only variables with correlations lower than 0.4. As we wanted only to reduce the number of predictor variables to test the hypothesis that social, climate and landscape factors have an effect on Hantavirus incidence on the Bayesian model, we always set up combinations of models containing climate, landscape and social variables (e.g., climate variables selected above; native vegetation cover, number of native vegetation fragments, native vegetation edge density; amount of corn, pasture and sugarcane; HDI, HDI elements; Gini index and poverty). Again, we used generalized linear mixed models, considering municipality and year as random effects, and the presence of HPS cases as the response variable. All estimated parameters were standardized, centered on their means and divided by two standard deviations. The model with the lower AIC were used in the Bayesian analysis (Table S1 shows the results of the best models of exploratory analysis for both regions).

Table S1. Exploratory analysis results made with generalized linear mixed model to reduce the number of predictor variables. Only the models that had an AIC value close to 2 are shown (PopRisk = population at risk; HDI = Human Development Index; PLAND = percent of habitat cover; NP = number of fragments; ED= habitat edge density; Cane = amount of sugarcane; Pasture = amount of pasture; TotalP= annual total precipitation; MaxP= annual maximum precipitation; MeanT = annual mean temperature; MeanTWint = annual mean temperature of winter).

| **Cerrado** | | | | | |
| --- | --- | --- | --- | --- | --- |
| Model name | Predictors Variables | AIC | Df | logLik | Deviance |
| m7 | PopRisk; HDI; PLAND;NP; Cane; TotalP; MeanT | 384.79 | 9 | -183.39 | 366.79 |
| m11 | PopRisk; HDI; NP; Cane; MaxP; MeanTWint | 385.04 | 9 | -183.52 | 367.04 |
| m5 | PopRisk; HDI; ED; Cane; MaxP; MeanT | 385.6 | 9 | -183.8 | 367.6 |
| m9 | PopRisk; HDI; ED; Cane; MaxP; MeanTWint | 385.73 | 9 | -183.87 | 367.73 |
| m6 | PopRisk; HDI; PLAND; Cane; MaxP; MeanT | 385.91 | 9 | -183.95 | 367.91 |
| m10 | PopRisk; HDI; PLAND; Cane; MaxP; MeanTWint | 386.2 | 9 | -184.1 | 368.2 |
| m31 | PopRisk; HDI; NP; Pasture; MaxP; MeanT | 386.73 | 9 | -184.36 | 368.73 |
| m4 | PopRisk; HDI; PLAND; NP; Cane; MaxP; MeanT | 386.73 | 10 | -183.37 | 366.73 |
| m8 | PopRisk; HDI; PLAND; NP; Cane; MaxP; MeanTWint | 386.95 | 10 | -183.47 | 366.95 |
| **Atlantic Forest** | | | | | |
| m7 | PopRisk; HDI; PLAND; NP; Cane; TotalP; MeanT | 982.55 | 9 | -482.28 | 964.55 |
| m3 | PopRisk; HDI; NP; Cane; TotalP; MeanT | 982.57 | 9 | -482.28 | 964.57 |
| m6 | PopRisk; HDI; PLAND; Cane; MaxP; MeanT | 983.95 | 9 | -482.97 | 965.95 |
| m4 | PopRisk; HDI; PLAND; NP; Cane; MaxP; MeanT | 984.04 | 10 | -482.02 | 964.04 |
| m2 | PopRisk; HDI; PLAND; Cane; TotalP; MeanT | 984.05 | 9 | -483.02 | 966.05 |
| M | PopRisk; HDI; NP; Cane; MaxP; MeanT | 984.1 | 10 | -482.05 | 964.1 |
| m1 | PopRisk; HDI; ED; Cane; TotalP; MeanT | 984.54 | 9 | -483.27 | 966.54 |
| m5 | PopRisk; HDI; ED; Cane; MaxP; MeanT | 984.8 | 9 | -483.4 | 966.8 |

References

Burnham KP, Anderson DR (2002) Model Selection and Multimodel Inference. A Practical Information—Theoretical Approach, Springer, New York
