# Supplementary material for: Landscape, Environmental and Social Predictors of Hantavirus Risk in São Paulo, Brazil
Source: PLoS One. 2016 Oct 25;11(10):e0163459. doi: 10.1371/journal.pone.0163459 (PMC5079598; doi:10.1371/journal.pone.0163459)
Supplement: S2 Table — (DOCX) [file pone.0163459.s002.docx]

Landscape, environmental and social predictors of Hantavirus risk in São Paulo, Brazil

Paula Ribeiro Prist^1*^, Maria Uriarte^2^, Leandro Reverberi Tambosi^1,2^, Amanda Prado^1^, Renata Pardini^3^, Paulo Sérgio D´Andrea^4^, Jean Paul Metzger^1^

**Supplementary** **Material**

**Exploratory analysis**

In both regions the same model was selected with the lower AIC. It was the model containing the variables population at risk, HDI, percent of habitat cover, number of fragments, amount of sugarcane, annual total precipitation and annual mean temperature (m7).

To be sure that our model contained all the best predictor variables, we also tested if the abundance of reservoir rodents of São Paulo had associations with Hantavirus incidence. To do so we extracted the abundance rodent data obtained in Prado (2015) in municipality levels (average abundance for each municipality for each reservoir rodent - *O. nigripes* and *N. lasiurus*).

Rodent abundance data were obtained through studies performed in Atlantic Forest region, conducted by Dra. Renata Pardini, Dra. Fabiana Umetsu and Dra. Adriana Bueno, from São Paulo University. The study area comprised areas of fragmented and continuously- forested landscapes characterized by different levels of remaining native forest (50, 30 and 10%), but similar with respect to climate, topography, type of forest and of human use, and distance to areas of continuous forest (Pardini et al. 2010). Small mammals were trapped with large pitfall traps in a total of 104 sampling points in 6 landscapes, of which 68 were collected in forest (18 in intact forest and 50 in forest fragments) and 36 in matrix areas. In control landscapes 6 points were sampled, being 3 in intact forest and 3 in intermediate forest. Greater details about point selection can be read in Bueno (2008), Umetsu (2010) and Puttker (2008). Total effort was 352 trap-night-point and 23.936 trap-night in the 68 points of the forest, and 176 trap-night-point, 6.336 trap-night in the 36 matrix points. Landscape metrics (percent of forest cover, edge density and percent of forest cover at landscape level) were extracted in ArcGis 10.3 buffers (200, 500 and 800 m) of each sampled point. Multiple regression models, in an information-theoretic approach was performed to find the best model, and the best buffer size. From the best model, selected through AIC, the abundance of *O. nigripes* and *N. lasiurus* were extrapolated from the sampled landscapes to the entire state of São Paulo, using the São Paulo state Forest Inventory map from 2010 (more details in Prado 2015).

Again, we used generalized linear mixed models, considering municipality and year as random effects, and the presence of HPS cases as the response variable. We performed a maximum likelihood model selection procedure, considering the second-order Akaike´s information criterion (AIC) (Burnham and Anderson 2002) to compare a set of 45 candidate models, for each biome, combining climate, social, landscape, agricultural and rodent variables, and including only variables with correlations lower than 0.4. Once rodent abundance data was extrapolated to São Paulo state based on landscape metrics, we never included abundance data and landscape metrics in the same model. All estimated parameters were standardized, centered on their means and divided by two standard deviations. The model with the lower AIC were used in the Bayesian analysis (Table S2 shows the results of the best models of exploratory analysis for both regions; for cerrado we also shown the first model with rodent included - once it does not appear in the best models selected).

Table S2. Exploratory analysis results made with generalized linear mixed model to reduce the number of predictor variables. Only the models that had an AIC value close to 2 are shown. For cerrado we also shown the first model with rodent included (once it does not appear in the best models selected). PopRisk = population at risk; HDI = Human Development Index; PLAND = percent of habitat cover; NP = number of fragments; ED= habitat edge density; Cane = amount of sugarcane; Pasture = amount of pasture; TotalP= annual total precipitation; MaxP= annual maximum precipitation; MeanT = annual mean temperature; MeanTWint = annual mean temperature of winter; NL = abundance of *Necromys lasiurus*; ON = abunandance of *Oligoryzomys nigripes*.

| **Cerrado** | | | | | |
| --- | --- | --- | --- | --- | --- |
| Model Nome | Predictors Variables | AIC | Df | logLik | Deviance |
| m7 | PopRisk; HDI; PLAND; NP; Cane; TotalP; MeanT | 384.79 | 9 | -183.39 | 366.79 |
| m11 | PopRisk; HDI; NP; Cane; MaxP; MeanTWint | 385.04 | 9 | -183.52 | 367.04 |
| m5 | PopRisk; HDI; ED; Cane; MaxP; MeanT | 385.6 | 9 | -183.8 | 367.6 |
| m9 | PopRisk; HDI; ED; Cane; MaxP; MeanTWint | 385.73 | 9 | -183.87 | 367.73 |
| m6 | PopRisk; HDI; PLAND; Cane; MaxP; MeanT | 385.91 | 9 | -183.95 | 367.91 |
| m10 | PopRisk; HDI; PLAND; Cane; MaxP; MeanTWint | 386.2 | 9 | -184.1 | 368.2 |
| m31 | PopRisk; HDI; NP; Pasture; MaxP; MeanT | 386.73 | 9 | -184.36 | 368.73 |
| m4 | PopRisk; HDI; PLAND; NP; Cane; MaxP; MeanT | 386.73 | 10 | -183.37 | 366.73 |
| m8 | PopRisk; HDI; PLAND; NP; Cane; MaxP; MeanTWint | 386.95 | 10 | -183.47 | 366.95 |
| r7 | PopRisk; HDI; NL; Pasture; MaxP; MeanT | 1360.84 | 10 | -670.42 | 1340.84 |
| **Atlantic Forest** | | | | | |
| m7 | PopRisk; HDI; PLAND; NP; Cane; TotalP; MeanT | 982.55 | 9 | -482.28 | 964.55 |
| m3 | PopRisk; HDI; NP; Cane; TotalP; MeanT | 982.57 | 9 | -482.28 | 964.57 |
| m6 | PopRisk; HDI; PLAND; Cane; MaxP; MeanT | 983.95 | 9 | -482.97 | 965.95 |
| m4 | PopRisk; HDI; PLAND; NP; Cane; MaxP; MeanT | 984.04 | 10 | -482.02 | 964.04 |
| m2 | PopRisk; HDI; PLAND; Cane; TotalP; MeanT | 984.05 | 9 | -483.02 | 966.05 |
| M | PopRisk; HDI; NP; Cane; MaxP; MeanT | 984.1 | 10 | -482.05 | 964.1 |
| r1 | PopRisk; HDI; ON; Cane; MaxP; MeanT | 984.51 | 9 | -483.25 | 966.51 |
| m1 | PopRisk; HDI; ED; Cane; TotalP; MeanT | 984.54 | 9 | -483.27 | 966.54 |
| m5 | PopRisk; HDI; ED; Cane; MaxP; MeanT | 984.8 | 9 | -483.4 | 966.8 |

Again, in both regions the model containing the predictor variables population at risk, HDI, percent of habitat cover, number of fragments, amount of sugarcane, annual total precipitation and annual mean temperature (m7) was selected with the lower AIC. In that way these variables were included in the final Bayesian model.

**References**

Bueno AA (2008) Pequenos mamíferos da Mata Atlântica do Planalto Atlântico paulista: uma avaliação da ameaça de extinção e da resposta a alterações no contexto e tamanho dos remanescentes. In: Instituto de Biociências. Departamento de Zoologia. Universidade de São Paulo, São Paulo, p. 124.

Pardini R, Bueno AA, Gardner TA, Prado PI, Metzger JP (2010) Beyond the Fragmentation Threshold Hypothesis: Regime Shifts in Biodiversity Across Fragmented Landscapes. PloSONE 10 e13666

Prado AF (2015) Abundância de roedores reservatórios de hantavírus no bioma da Mata Atlântica: efeitos da estrutura da paisagem e da escala de análise. In: Instituto de Biociências. Departamento de Ecologia. Universidade de São Paulo, São Paulo, p. 46.

Puttker T, Pardini R, Meyer-Lucht Y, Sommer S. Responses of five small mammal species to micro-scale variations in vegetation structure in secondary Atlantic Forest remnants, Brazil. 2008b; BMC Ecology 8:9. http://www.biomedcentral.com/1472-6785/8/9

Umetsu U, Metzger JP, Pardini R (2008) Importance of estimating matrix quality for modeling species distribution in complex tropical landscapes: a test with Atlantic forest small mammals. Ecography 31: 359-370
