# Supplementary material for: Landscape, Environmental and Social Predictors of Hantavirus Risk in São Paulo, Brazil
Source: PLoS One. 2016 Oct 25;11(10):e0163459. doi: 10.1371/journal.pone.0163459 (PMC5079598; doi:10.1371/journal.pone.0163459)
Supplement: S3 Table — (DOCX) [file pone.0163459.s003.docx]

Landscape, environmental and social predictors of Hantavirus risk in São Paulo, Brazil

Paula Ribeiro Prist^1*^, Maria Uriarte^2^, Leandro Reverberi Tambosi^1,2^, Amanda Prado^1^, Renata Pardini^3^, Paulo Sérgio D´Andrea^4^, Jean Paul Metzger^1^

**Supplementary** **Material**

All set of candidate models analyzed in the generalized linear mixed models are shown in table S.3.

Table S3. All set of candidate models analyzed in the generalized linear mixed models.

| Model Name | Predictor Variables |
| --- | --- |
| t1 | Annual mean precipitation |
| t2 | Annual total precipitation (A) |
| t3 | Annual minimum precipitation |
| t4 | Annual maximum precipitation (B) |
| t5 | Annual mean precipitation of summer |
| t6 | Annual minimum precipitation of summer |
| t7 | Annual maximum precipitation of summer |
| t8 | Annual mean precipitation of fall |
| t9 | Annual minimum precipitation of fall |
| t10 | Annual maximum precipitation of fall |
| t11 | Annual mean precipitation of winter |
| t12 | Annual minimum precipitation of winter |
| t13 | Annual maximum precipitation of winter |
| t14 | Annual mean precipitation of spring |
| t15 | Annual minimum precipitation of spring |
| t16 | Annual maximum precipitation of spring |
| t17 | Annual mean temperature (C) |
| t18 | Annual mean temperature of summer |
| t19 | Annual mean temperature of fall |
| t20 | Annual mean temperature of winter (D) |
| t21 | Annual mean temperature of spring |
| M | Population at risk (E), HDI (F), Number of fragments, Sugarcane; (B) and (C) |
| m1 | (E), (F), Edge density, sugarcane, (A), (C) |
| m2 | (E), (F), Percent of habitat cover, sugarcane, (A), (C) |
| m3 | (E), (F), Number of fragments, sugarcane, (A), (C) |
| m4 | (E), (F), Percent of habitat cover, Number of fragments, sugarcane, (B), (C) |
| m5 | (E), (F), Edge density, sugarcane, (B), (C) |
| m6 | (E), (F), Percent of habitat cover, sugarcane, (B), (C) |
| m7 | (E), (F), Percent of habitat cover, Number of fragments, sugarcane; (A), (C) |
| m8 | (E), (F), Percent of habitat cover, Number of fragments, sugarcane; (B), (D) |
| m9 | (E), (F), Edge density; sugarcane, (B), (D) |
| m10 | (E), (F), Percent of habitat cover, sugarcane, (B), (D) |
| m11 | (E), (F), Number of fragments, sugarcane, (B), (D) |
| m12 | (E), (F), Percent of habitat cover, Number of fragments, Corn, (A), (C) |
| m13 | (E), (F), Edge density, Corn, (A), (C) |
| m14 | (E), (F), Percent of habitat cover, Corn, (A), (C) |
| m15 | (E), (F), Number of fragments, Corn, (A), (C) |
| m16 | (E), (F), Percent of habitat cover, Number of fragments, Corn, (B), (C) |
| m17 | (E), (F), Edge density, Corn, (B), (C) |
| m18 | (E), (F), Percent of habitat cover, Corn, (B), (C) |
| m19 | (E), (F), Number of fragments, Corn, (B), (C) |
| m20 | (E), (F), Percent of habitat cover, Number of fragments, Corn, (B), (D) |
| m21 | (E), (F), Edge density, Corn, (B), (D) |
| m22 | (E), (F), Percent of habitat cover, Corn, (B), (D) |
| m23 | (E), (F), Number of fragments, Corn, (B), (D) |
| m24 | (E), (F), Percent of habitat cover, Number of fragments, Pasture, (A), (C) |
| m25 | (E), (F), Edge density, Pasture, (A), (C) |
| m26 | (E), (F), Percent of habitat cover, Pasture, (A), (C) |
| m27 | (E), (F), Number of fragments, Pasture, (A), (C) |
| m28 | (E), (F), Percent of habitat cover, Number of fragments, Pasture, (B), (C) |
| m29 | (E), (F), Edge density, Pasture, (B), (C) |
| m30 | (E), (F), Percent of habitat cover, Pasture, (B), (C) |
| m31 | (E), (F), Number of fragments, Pasture, (B), (C) |
| m32 | (E), (F), Percent of habitat cover, Number of fragments, Pasture, (B), (D) |
| m33 | (E), (F), Edge density, Pasture, (B), (D) |
| m34 | (E), (F), Percent of habitat cover, Pasture, (B), (D) |
| m35 | (E), (F), Number of fragments, Pasture, (B), (D) |
| Mg | (E), Gini, Number of fragments, sugarcane, (B), (C) |
| mg1 | (E), Gini, Edge density, sugarcane, (B), (C) |
| mg2 | (E), Gini, Percent of habitat cover, sugarcane, (B), (C) |
| mg3 | (E), Gini, Number of fragments, sugarcane, (A), (C) |
| mg4 | (E), Gini, Percent of habitat cover, Number of fragments, sugarcane, (B), (C) |
| mg5 | (E), Gini, Edge density, sugarcane, (B), (C) |
| mg6 | (E), Gini, Percent of habitat cover, sugarcane, (B), (C) |
| mg7 | (E), Gini, Percent of habitat cover, Number of fragments, sugarcane, (A), (C) |
| mg8 | (E), Gini, Percent of habitat cover, Number of fragments, sugarcane; (B), (D) |
| mg9 | (E), Gini, Edge density, sugarcane, (B), (D) |
| mg10 | (E), Gini, Percent of habitat cover, sugarcane, (B), (D) |
| mg11 | (E), Gini, Number of fragments, sugarcane, (B), (D) |
| mg12 | (E), Gini, Percent of habitat cover, Number of fragments, Corn, (A), (C) |
| mg13 | (E), Gini, Edge density, Corn, (A), (C) |
| mg14 | (E), Gini, Percent of habitat cover, Corn, (A), (C) |
| mg15 | (E), Gini, Number of fragments, Corn, (A), (C) |
| mg16 | (E), Gini, Percent of habitat cover, Number of fragments, Corn, (B), (C) |
| mg17 | (E), Gini, Edge density, Corn, (B), (C) |
| mg18 | (E), Gini, Percent of habitat cover, Corn, (B), (C) |
| mg19 | (E), Gini, Number of fragments, Corn, (B), (C) |
| mg20 | (E), Gini, Percent of habitat cover, Number of fragments, Corn, (B), (D) |
| mg21 | (E), Gini, Edge density, Corn, (B), (D) |
| mg22 | (E), Gini, Percent of habitat cover, Corn, (B), (D) |
| mg23 | (E), Gini, Number of fragments, Corn, (B), (D) |
| mg24 | (E), Gini, Percent of habitat cover, Number of fragments, Pasture, (A), (C) |
| mg25 | (E), Gini, Edge density, Pasture, (A), (C) |
| mg26 | (E), Gini, Percent of habitat cover, Pasture, (A), (C) |
| mg27 | (E), Gini, Number of fragments, Pasture, (A), (C) |
| mg28 | (E), Gini, Percent of habitat cover, Number of fragments, Pasture, (B), (C) |
| mg29 | (E), Gini, Edge density, Pasture, (B), (C) |
| mg30 | (E), Gini, Percent of habitat cover, Pasture, (B), (C) |
| mg31 | (E), Gini, Number of fragments, Pasture, (B), (C) |
| mg32 | (E), Gini, Percent of habitat cover, Number of fragments, Pasture, (B), (D) |
| mg33 | (E), Gini, Edge density, Pasture, (B), (D) |
| mg34 | (E), Gini, Percent of habitat cover, Pasture, (B), (D) |
| mg35 | (E), Gini, Number of fragments, Pasture, (B), (D) |
| Mp | (E), Poverty, Number of fragments, sugarcane, (B), (C) |
| mp1 | (E), Poverty, Edge density, sugarcane, (A), (C) |
| mp2 | (E), Poverty, Percent of habitat cover, sugarcane, (A), (C) |
| mp3 | (E), Poverty, Number of fragments, sugarcane, (A), (C) |
| mp4 | (E), Poverty, Percent of habitat cover, Number of fragments, sugarcane, (B), (C) |
| mp5 | (E), Poverty, Edge density, sugarcane, (B), (C) |
| mp6 | (E), Poverty, Percent of habitat cover, sugarcane, (B), (C) |
| mp7 | (E), Poverty, Percent of habitat cover, Number of fragments, sugarcane, (A), (C) |
| mp8 | (E), Poverty, Percent of habitat cover, Number of fragments, sugarcane, (B), (D) |
| mp9 | (E), Poverty, Edge density, sugarcane, (B), (D) |
| mp10 | (E), Poverty, Percent of habitat cover, sugarcane, (B), (D) |
| mp11 | (E), Poverty, Number of fragments, sugarcane, (B), (D) |
| mp12 | (E), Poverty, Percent of habitat cover, Number of fragments, Corn, (A), (C) |
| mp13 | (E), Poverty, Edge density, Corn, (A), (C) |
| mp14 | (E), Poverty, Percent of habitat cover, Corn, (A), (C) |
| mp15 | (E), Poverty, Number of fragments, Corn, (A), (C) |
| mp16 | (E), Poverty, Percent of habitat cover, Number of fragments, Corn (B), (C) |
| mp17 | (E), Poverty, Edge density, Corn, (B), (C) |
| mp18 | (E), Poverty, Percent of habitat cover, Corn, (B), (C) |
| mp19 | (E), Poverty, Number of fragments, Corn, (B), (C) |
| mp20 | (E), Poverty, Percent of habitat cover, Number of fragments, Corn, (B), (D) |
| mp21 | (E), Poverty, Edge density, Corn, (B), (D) |
| mp22 | (E), Poverty, Percent of habitat cover, Corn, (B), (D) |
| mp23 | (E), Poverty, Number of fragments, Corn, (B), (D) |
| mp24 | (E), Poverty, Percent of habitat cover, Number of fragments, Pasture, (A), (C) |
| mp25 | (E), Poverty, Edge density, Pasture, (A), (C) |
| mp26 | (E), Poverty, Percent of habitat cover, Pasture, (A), (C) |
| mp27 | (E), Poverty, Number of fragments, Pasture, (A), (C) |
| mp28 | (E), Poverty, Percent of habitat cover, Number of fragments, Pasture, (B), (C) |
| mp29 | (E), Poverty, Edge density, Pasture, (B), (C) |
| mp30 | (E), Poverty, Percent of habitat cover, Pasture, (B), (C) |
| mp31 | (E), Poverty, Number of fragments, Pasture, (B), (C) |
| mp32 | (E), Poverty, Percent of habitat cover, Number of fragments, Pasture, (B), (D) |
| mp33 | (E), Poverty, Edge density, Pasture, (B), (D) |
| mp34 | (E), Poverty, Percent of habitat cover, Pasture, (B), (D) |
| mp35 | (E), Poverty, Number of fragments, Pasture, (B), (D) |
| Mi | (E), Income, Number of fragments, sugarcane, (B), (C) |
| mi1 | (E), Income, Edge density, sugarcane, (A), (C) |
| mi2 | (E), Income, Percent of habitat cover, sugarcane, (A), (C) |
| mi3 | (E), Income, Number of fragments, sugarcane, (A), (C) |
| mi4 | (E), Income, Percent of habitat cover, Number of fragments, sugarcane, (B), (C) |
| mi5 | (E), Income, Edge density, sugarcane, (B), (C) |
| mi6 | (E), Income, Percent of habitat cover, sugarcane, (B), (C) |
| mi7 | (E), Income, Percent of habitat cover, Number of fragments, sugarcane, (A), (C) |
| mi8 | (E), Income, Percent of habitat cover, Number of fragments, sugarcane, (B), (D) |
| mi9 | (E), Income, Edge density, sugarcane, (B), (D) |
| mi10 | (E), Income, Percent of habitat cover, sugarcane, (B), (D) |
| mi11 | (E), Income, Number of fragments, sugarcane, (B), (D) |
| mi12 | (E), Income, Percent of habitat cover, Number of fragments, Corn, (A), (C) |
| mi13 | (E), Income, Edge density, Corn, (A), (C) |
| mi14 | (E), Income, Percent of habitat cover, Corn, (A), (C) |
| mi15 | (E), Income, Number of fragments, Corn, (A), (C) |
| mi16 | (E), Income, Percent of habitat cover, Number of fragments, Corn, (B), (C) |
| mi17 | (E), Income, Edge density, Corn, (B), (C) |
| mi18 | (E), Income, Percent of habitat cover, Corn, (B), (C) |
| mi19 | (E), Income, Number of fragments, Corn, (B), (C) |
| mi20 | (E), Income, Percent of habitat cover, Number of fragments, Corn, (B), (D) |
| mi21 | (E), Income, Edge density, Corn, (B), (D) |
| mi22 | (E), Income, Percent of habitat cover, Corn, (B), (D) |
| mi23 | (E), Income, Number of fragments, Corn, (B), (D) |
| mi24 | (E), Income, Percent of habitat cover, Number of fragments, Pasture, (A), (C) |
| mi25 | (E), Income, Edge density, Pasture, (A), (C) |
| mi26 | (E), Income, Percent of habitat cover, Pasture, (A), (C) |
| mi27 | (E), Income, Number of fragments, Pasture, (A), (C) |
| mi28 | (E), Income, Percent of habitat cover, Number of fragments, Pasture, (B), (C) |
| mi29 | (E), Income, Edge density, Pasture, (B), (C) |
| mi30 | (E), Income; Percent of habitat cover , Pasture, (B), (C) |
| mi31 | (E), Income, Number of fragments, Pasture, (B), (C) |
| mi32 | (E), Income; Percent of habitat cover, Number of fragments, Pasture, (B), (D) |
| mi33 | (E), Income, Edge density, Pasture, (B), (D) |
| mi34 | (E), Income, Percent of habitat cover, Pasture, (B), (D) |
| mi35 | (E), Income, Number of fragments, Pasture, (B), (D) |
| Ml | (E), Life_expectancy, Number of fragments, sugarcane, (B), (C) |
| ml1 | (E), Life_expectancy, Edge density, sugarcane, (A), (C) |
| ml2 | (E), Life_expectancy, Percent of habitat cover, sugarcane, (A), (C) |
| ml3 | (E), Life_expectancy, Number of fragments, sugarcane, (A), (C) |
| ml4 | (E), Life_expectancy, Percent of habitat cover, Number of fragments, sugarcane, (B), (C) |
| ml5 | (E), Life_expectancy, Edge density, sugarcane, (B), (C) |
| ml6 | (E), Life_expectancy, Percent of habitat cover, sugarcane, (A), (C) |
| ml7 | (E), Life_expectancy, Percent of habitat cover, Number of fragments, sugarcane, (A), (C) |
| ml8 | (E), Life_expectancy, Percent of habitat cover, Number of fragments, sugarcane, (B), (D) |
| ml9 | (E), Life_expectancy, Edge density, sugarcane, (B), (D) |
| ml10 | (E), Life_expectancy, Percent of habitat cover, sugarcane, (B), (D) |
| ml11 | (E), Life_expectancy, Number of fragments, sugarcane, (B), (D) |
| ml12 | (E), Life_expectancy, Percent of habitat cover, Number of fragments, Corn, (A), (C) |
| ml13 | (E), Life_expectancy, Edge density, Corn, (A), (C) |
| ml14 | (E), Life_expectancy, Percent of habitat cover, Corn, (A), (C) |
| ml15 | (E), Life_expectancy, Number of fragments, Corn, (A), (C) |
| ml16 | (E), Life_expectancy, Percent of habitat cover, Number of fragments, Corn, (B), (C) |
| ml17 | (E), Life_expectancy, Edge density, Corn, (B), (C) |
| ml18 | (E), Life_expectancy, Percent of habitat cover, Corn, (B), (C) |
| ml19 | (E), Life_expectancy, Number of fragments, Corn, (B), (C) |
| ml20 | (E), Life_expectancy, Percent of habitat cover, Number of fragments, Corn, (B), (D) |
| ml21 | (E), Life_expectancy, Edge density, Corn, (B), (D) |
| ml22 | (E), Life_expectancy, Percent of habitat cover, Corn; (B), (D) |
| ml23 | (E), Life_expectancy, Number of fragments, Corn, (B), (D) |
| ml24 | (E), Life_expectancy, Percent of habitat cover, Number of fragments, Pasture, (A), (C) |
| ml25 | (E), Life_expectancy, Edge density, Pasture, (A), (C) |
| ml26 | (E), Life_expectancy, Percent of habitat cover, Pasture, (A), (C) |
| ml27 | (E), Life_expectancy, Number of fragments, Pasture, (A), (C) |
| ml28 | (E), Life_expectancy, Percent of habitat cover, Number of fragments, Pasture, (B), (C) |
| ml29 | (E), Life_expectancy, Edge density, Pasture, (B), (C) |
| ml30 | (E), Life_expectancy, Percent of habitat cover, Pasture, (B), (C) |
| ml31 | (E), Life_expectancy, Number of fragments, Pasture, (B), (C) |
| ml32 | (E), Life_expectancy, Percent of habitat cover, Number of fragments, Pasture; (B), (D) |
| ml33 | (E), Life_expectancy, Edge density, Pasture, (B), (D) |
| ml34 | (E), Life_expectancy, Percent of habitat cover, Pasture; (B), (D) |
| ml35 | (E), Life_expectancy, Number of fragments, Pasture; (B), (D) |
| Me | (E), education, Number of fragments, sugarcane (B), (C) |
| me1 | (E), education, Edge density, sugarcane, (A), (C) |
| me2 | (E), education, Percent of habitat cover, sugarcane, (A), (C) |
| me3 | (E), education, Number of fragments, sugarcane, (A), (C) |
| me4 | (E), education, Percent of habitat cover, Number of fragments, sugarcane, (B), (C) |
| me5 | (E), education, Edge density, sugarcane, (B), (C) |
| me6 | (E), education, Percent of habitat cover, sugarcane, (B), (C) |
| me7 | (E), education, Percent of habitat cover, Number of fragments, sugarcane, (A), (C) |
| me8 | (E), education, Percent of habitat cover, Number of fragments, sugarcane, (B), (D) |
| me9 | (E), education, Edge density, sugarcane, (B), (D) |
| me10 | (E), education, Percent of habitat cover, sugarcane, (B), (D) |
| me11 | (E), education, Number of fragments, sugarcane, (B), (D) |
| me12 | (E), education, Percent of habitat cover, Number of fragments, Corn, (A), (C) |
| me13 | (E), education, Edge density, Corn, (A), (C) |
| me14 | (E), education, Percent of habitat cover, Corn, (A), (C) |
| me15 | (E), education, Number of fragments, Corn, (A), (C) |
| me16 | (E), education, Percent of habitat cover, Number of fragments, Corn, (B), (C) |
| me17 | (E), education, Edge density, Corn, (B), (C) |
| me18 | (E), education, Percent of habitat cover, Corn, (B), (C) |
| me19 | (E), education, Number of fragments, Corn, (B), (C) |
| me20 | (E), education, Percent of habitat cover, Number of fragments, Corn, (B), (D) |
| me21 | (E), education, Edge density, Corn, (B), (D) |
| me22 | (E), education, Percent of habitat cover, Corn, (B), (D) |
| me23 | (E), education, Number of fragments, Corn, (B), (D) |
| me24 | (E), education, Percent of habitat cover, Number of fragments, Pasture, (A), (C) |
| me25 | (E), education, Edge density, Pasture, (A), (C) |
| me26 | (E), education, Percent of habitat cover, Pasture, (A), (C) |
| me27 | (E), education, Number of fragments, Pasture, (A), (C) |
| me28 | (E), education, Percent of habitat cover, Number of fragments, Pasture, (B), (C) |
| me29 | (E), education, Edge density, Pasture, (B), (C) |
| me30 | (E), education, Percent of habitat cover, Pasture; (B), (C) |
| me31 | (E), education, Number of fragments, Pasture, (B), (C) |
| me32 | (E), education, Percent of habitat cover, Number of fragments, Pasture, (B), (D) |
| me33 | (E), education, Edge density, Pasture, (B), (D) |
| me34 | (E), education, Percent of habitat cover, Pasture, (B), (D) |
| me35 | (E), education, Number of fragments, Pasture, (B), (D) |
| R | (E), (F)/Gini/poverty/income/life-expectancy/education, NL/ON, sugarcane, (A), (C) |
| r1 | (E), (F) Gini/poverty/income/life-expectancy/education, NL/ON, sugarcane; (B), (C) |
| r2 | (E), (F) Gini/poverty/income/life-expectancy/education, NL/ON, sugarcane, (B), (D) |
| r3 | (E),(F) Gini/poverty/income/life-expectancy/education, NL/ON, Corn, (A), (C) |
| r4 | (E), (F) Gini/poverty/income/life-expectancy/education, NL/ON, Corn, (B), (C) |
| r5 | (E), (F) Gini/poverty/income/life-expectancy/education, NL/ON, Corn, (B), (D) |
| r6 | (E), (F) Gini/poverty/income/life-expectancy/education, NL/ON, Pasture, (A), (C) |
| r7 | (E), (F) Gini/poverty/income/life-expectancy/education, NL/ON, Pasture, (B), (C) |
| r8 | (E), (F) Gini/poverty/income/life-expectancy/education, NL/ON, Pasture, (B), (D) |
