# Supplementary material for: Landscape, Environmental and Social Predictors of Hantavirus Risk in São Paulo, Brazil
Source: PLoS One. 2016 Oct 25;11(10):e0163459. doi: 10.1371/journal.pone.0163459 (PMC5079598; doi:10.1371/journal.pone.0163459)
Supplement: S4 Table — (DOCX) [file pone.0163459.s004.docx]

Landscape, environmental and social predictors of Hantavirus risk in São Paulo, Brazil

Paula Ribeiro Prist^1*^, Maria Uriarte^2^, Leandro Reverberi Tambosi^1,2^, Amanda Prado^1^, Renata Pardini^3^, Paulo Sérgio D´Andrea^4^, Jean Paul Metzger^1^

**Supplementary** **Material**

Table S4. Predictor variables included in the model

| *Predictor variable* | *Description* | *Years available* | *Source data* |
| --- | --- | --- | --- |
| People at risk | Number of men older than 14 years employed/ living in agricultural areas | 1996/2006 | IBGE |
| HDI | Human Development Index | 1991/2000/2010 | IBGE |
| Forest | Percentage of native vegetation cover in municipalities | 2000/2010 | São Paulo state Forest Inventory |
| NP | Number of native vegetation patches in municipalities | 2000/2010 | São Paulo state Forest Inventory |
| Sugarcane | Percent of municipality occupied by sugarcane | 1993 to 2012 | Agricultural Census of Institute of Agricultural Economics |
| Total Precipitation | Total annual precipitation | 1993 to 2012 | Climate Hazards Group Infrared Precipitation with Stations |
| Mean Temperature | Mean annual temperature per year | 1993 to 2012 | National Centers for Environmental Prediction |
