# Supplementary material for: Landscape, Environmental and Social Predictors of Hantavirus Risk in São Paulo, Brazil
Source: PLoS One. 2016 Oct 25;11(10):e0163459. doi: 10.1371/journal.pone.0163459 (PMC5079598; doi:10.1371/journal.pone.0163459)
Supplement: S5 Table — (DOCX) [file pone.0163459.s005.docx]

Landscape, environmental and social predictors of Hantavirus risk in São Paulo, Brazil

Paula Ribeiro Prist^1*^, Maria Uriarte^2^, Leandro Reverberi Tambosi^1,2^, Amanda Prado^1^, Renata Pardini^3^, Paulo Sérgio D´Andrea^4^, Jean Paul Metzger^1^

**Supplementary** **Material**

Table S5. Moran´s I test applied to the residuals of the Bernoulli models for cerrado (Table S5A) and Atlantic forest region (Table S5B) of São Paulo State. Moran´s I Test was performed for each year we had data (1993 - 2012), and used the spatial contiguity matrix based on the Queen´s case neighborhood relation. *: significant result.

| *(A) cerrado region* | | |
| --- | --- | --- |
| Year | Moran I statistic | p-value |
| 1993 | 0.0891 | 0.0443* |
| 1994 | -0.015692173 | 0.5923 |
| 1995 | -0.014679862 | 0.5743 |
| 1996 | -0.018715981 | 0.6113 |
| 1997 | -0.013574461 | 0.5471 |
| 1998 | -0.024832829 | 0.6523 |
| 1999 | -0.005903901 | 0.256 |
| 2000 | -0.0519536464 | 0.729 |
| 2001 | -0.037687182 | 0.717 |
| 2002 | 0.008955163 | 0.3917 |
| 2003 | 0.015124948 | 0.3521 |
| 2004 | -0.096034078 | 0.913 |
| 2005 | -0.076794304 | 0.8752 |
| 2006 | 0.055305607 | 0.1102 |
| 2007 | -0.019493992 | 0.5687 |
| 2008 | 0.007485780 | 0.4118 |
| 2009 | -0.046907146 | 0.7303 |
| 2010 | -0.051158416 | 0.7268 |
| 2011 | -0.023866604 | 0.5942 |
| 2012 | -0.029805356 | 0.6324 |

| *(B) Atlantic forest Region* | | |
| --- | --- | --- |
| Year | Moran I statistic | p-value |
| 1993 | 0.0077140407 | 0.263 |
| 1994 | -0.0315037475 | 0.8504 |
| 1995 | 0.0173919765 | 0.2463 |
| 1996 | -0.0085926427 | 0.6139 |
| 1997 | -0.0298241028 | 0.8349 |
| 1998 | -0.0050288202 | 0.5456 |
| 1999 | -0.0209857310 | 0.7429 |
| 2000 | -0.0073396152 | 0.5848 |
| 2001 | -0.0017712202 | 0.4954 |
| 2002 | -0.0151332196 | 0.6727 |
| 2003 | -0.0253334696 | 0.7834 |
| 2004 | -0.0064223055 | 0.5589 |
| 2005 | -0.0071336002 | 0.5698 |
| 2006 | -0.0099091010 | 0.6067 |
| 2007 | -0.0216753551 | 0.7446 |
| 2008 | -0.0074002665 | 0.5707 |
| 2009 | 0.0529313912 | 0.03095* |
| 2010 | -0.023258202 | 0.7621 |
| 2011 | -0.0251251082 | 0.7802 |
| 2012 | -0.0334661104 | 0.8626 |
