# Supplementary material for: Landscape, Environmental and Social Predictors of Hantavirus Risk in São Paulo, Brazil
Source: PLoS One. 2016 Oct 25;11(10):e0163459. doi: 10.1371/journal.pone.0163459 (PMC5079598; doi:10.1371/journal.pone.0163459)
Supplement: S6 Table — (DOCX) [file pone.0163459.s006.docx]

Landscape, environmental and social predictors of Hantavirus risk in São Paulo, Brazil

Paula Ribeiro Prist^1*^, Maria Uriarte^2^, Leandro Reverberi Tambosi^1,2^, Amanda Prado^1^, Renata Pardini^3^, Paulo Sérgio D´Andrea^4^, Jean Paul Metzger^1^

**Supplementary** **Material**

Table S6. Moran´s I test applied to the number of HPS cases for cerrado (Table S6A) and Atlantic forest region (Table S6B) of São Paulo State. Moran´s I Test was performed for each year we had data (1993 - 2012) and used the spatial contiguity matrix based on the Queen´s case neighborhood relation. *: significant result. Empty spaces means years with no diseases cases.

| *(A) cerrado region* | | |
| --- | --- | --- |
| Year | Moran I statistic | p-value |
| 1993 |  |  |
| 1994 |  |  |
| 1995 |  |  |
| 1996 |  |  |
| 1997 |  |  |
| 1998 | -0.02002 | 0.7803 |
| 1999 | -0.02002 | 0.7803 |
| 2000 | -0.00967 | 0.645 |
| 2001 | -0.00487 | 0.477 |
| 2002 | -0.00439 | 0.46 |
| 2003 | -0.00652 | 0.491 |
| 2004 | -0.05601 | 0.7698 |
| 2005 | -0.02008 | 0.5998 |
| 2006 | 0.000093 | 0.089 |
| 2007 | -0.00611 | 0.4904 |
| 2008 | -0.01426 | 0.5463 |
| 2009 | -0.00652 | 0.491 |
| 2010 | -0.01642 | 0.550 |
| 2011 | -0.00696 | 0.4953 |
| 2012 | -0.00480 | 0.4814 |

| *(B)Atlantic Forest Region* | | |
| --- | --- | --- |
| Year | Moran I statistic | p-value |
| 1993 | -00004.70 | 0.065 |
| 1994 |  |  |
| 1995 |  |  |
| 1996 | -0.00653 | 0.58 |
| 1997 |  |  |
| 1998 | -0.00449 | 0.537 |
| 1999 | -0.01885 | 0.7286 |
| 2000 | -0.0099 | 0.9231 |
| 2001 | -0.0139 | 0.6611 |
| 2002 | -0.0126 | 0.6531 |
| 2003 | -0.0164 | 0.7293 |
| 2004 | -0.0127 | 0.6477 |
| 2005 | -0.01299 | 0.6578 |
| 2006 | -0.01147 | 0.6368 |
| 2007 | -0.01096 | 0.634 |
| 2008 | 0.01208 | 0.3167 |
| 2009 | 0.0429 | 0.0626 |
| 2010 | -0.0302 | 0.8484 |
| 2011 | -0.0302 | 0.8421 |
| 2012 | -0.00871 | 0.6071 |
