# Supplementary material for: Landscape, Environmental and Social Predictors of Hantavirus Risk in São Paulo, Brazil
Source: PLoS One. 2016 Oct 25;11(10):e0163459. doi: 10.1371/journal.pone.0163459 (PMC5079598; doi:10.1371/journal.pone.0163459)
Supplement: S7 Table — (DOCX) [file pone.0163459.s007.docx]

Landscape, environmental and social predictors of Hantavirus risk in São Paulo, Brazil

Paula Ribeiro Prist^1*^, Maria Uriarte^2^, Leandro Reverberi Tambosi^1,2^, Amanda Prado^1^, Renata Pardini^3^, Paulo Sérgio D´Andrea^4^, Jean Paul Metzger^1^

**Supplementary** **Material**

Table S7. Average and range values of annual mean temperature and total precipitation for the municipalities of cerrado and Atlantic Forest regions, from 1993 to 2012.

|  | Cerrado |  | Atlantic Forest |  |
| --- | --- | --- | --- | --- |
| Year | Mean Temperature (°C) | Total Precipitation (mm) | Mean Temperature (°C) | Total Precipitation (mm) |
| 1993 | 22.48 (18.8-24.22) | 1340 (1089-2062) | 22.49 (18.7-24.4) | 1358 (1000-2338) |
| 1994 | 22.73 (18.7-24.9) | 1194 (948-1917) | 22.77 (18.5-24.9) | 1227 (922-2390) |
| 1995 | 23.31 (18.6-25.5) | 1411 (1170-2461) | 23.25 (18.3- 25.6) | 1443 (1115-2895) |
| 1996 | 23.16 (19.1-25.2) | 1389 (1053-2368) | 23.17 (19-25.3) | 1426 (1022-2728) |
| 1997 | 22.86 (18.7-24.7) | 1356 (1161-1891) | 22.80 (18.4-24.7) | 1396 (1103-2275) |
| 1998 | 22.97 (19.1- 24.6) | 1352 (1178-2171) | 22.91 (18.8-24.6) | 1400 (1150-2728) |
| 1999 | 23.15 (19 - 25.1) | 1187 (831-2087) | 23.07 (18.9-25.1) | 1186 (804-2325) |
| 2000 | 22.71 (18.3 - 25) | 1339 (1057-2244) | 22.63 (18-25.2) | 1356 (1053-2522) |
| 2001 | 22.90 (19 - 25) | 1414 (1148-2131) | 22.76 (18.6-25.1) | 1438 (1147-2426) |
| 2002 | 23.01 (19.4- 25.1) | 1270 (927-1891) | 23.01 (19.3-25.2) | 1286 (883-2349) |
| 2003 | 23.87 (19.9-26.5) | 1261 (915-1799) | 23.86 (19.7-26.7) | 1275 (892-2031) |
| 2004 | 22.83 (19 - 25) | 1204 (927-1983) | 22.83 (18.7-25.2) | 1235 (909-2310) |
| 2005 | 22.44 (18.3-25.7) | 1279 (1032-2169) | 22.48 (18.2-26-2) | 1333 (1018-2559) |
| 2006 | 22.86 (18.5-26) | 1312 (1101-1910) | 22.90 (18.4-26.4) | 1332 (1083-2309) |
| 2007 | 23.05 (18.8-26) | 1273 (1051-2058) | 23.14 (18.8-27.6) | 1315 (1038-2534) |
| 2008 | 23.11 (18.8-26.7) | 1219 (970-2490) | 23.17 (18.7-27.6) | 1302 (957-3079) |
| 2009 | 22.91 (18.7-26) | 1652 (1383-3218) | 22.87 (18.5-26) | 1729 (1322-3936) |
| 2010 | 22.74 (19 - 24.9) | 1188 (895-2637) | 22.68 (18.8- 24.9) | 1282 (846-3275) |
| 2011 | 22.95 (19-24.4) | 1384 (1139-2227) | 22.81 (19-24.5) | 1414 (1120-2703) |
| 2012 | 22.67 (18.77-24.48) | 1452 (1066-2631) | 22.57 (18.7-24.4) | 1483 (1068-3093) |
