# Supplementary material for: Landscape, Environmental and Social Predictors of Hantavirus Risk in São Paulo, Brazil
Source: PLoS One. 2016 Oct 25;11(10):e0163459. doi: 10.1371/journal.pone.0163459 (PMC5079598; doi:10.1371/journal.pone.0163459)
Supplement: S1 Fig — Amount of native vegetation (A) and sugar cane plantation (B) in the state of São Paulo. (DOCX) [file pone.0163459.s008.docx]

Landscape, environmental and social predictors of Hantavirus risk in São Paulo, Brazil

Paula Ribeiro Prist^1*^, Maria Uriarte^2^, Leandro Reverberi Tambosi^1,2^, Amanda Prado^1^, Renata Pardini^3^, Paulo Sérgio D´Andrea^4^, Jean Paul Metzger^1^

**Supplementary** **Material**


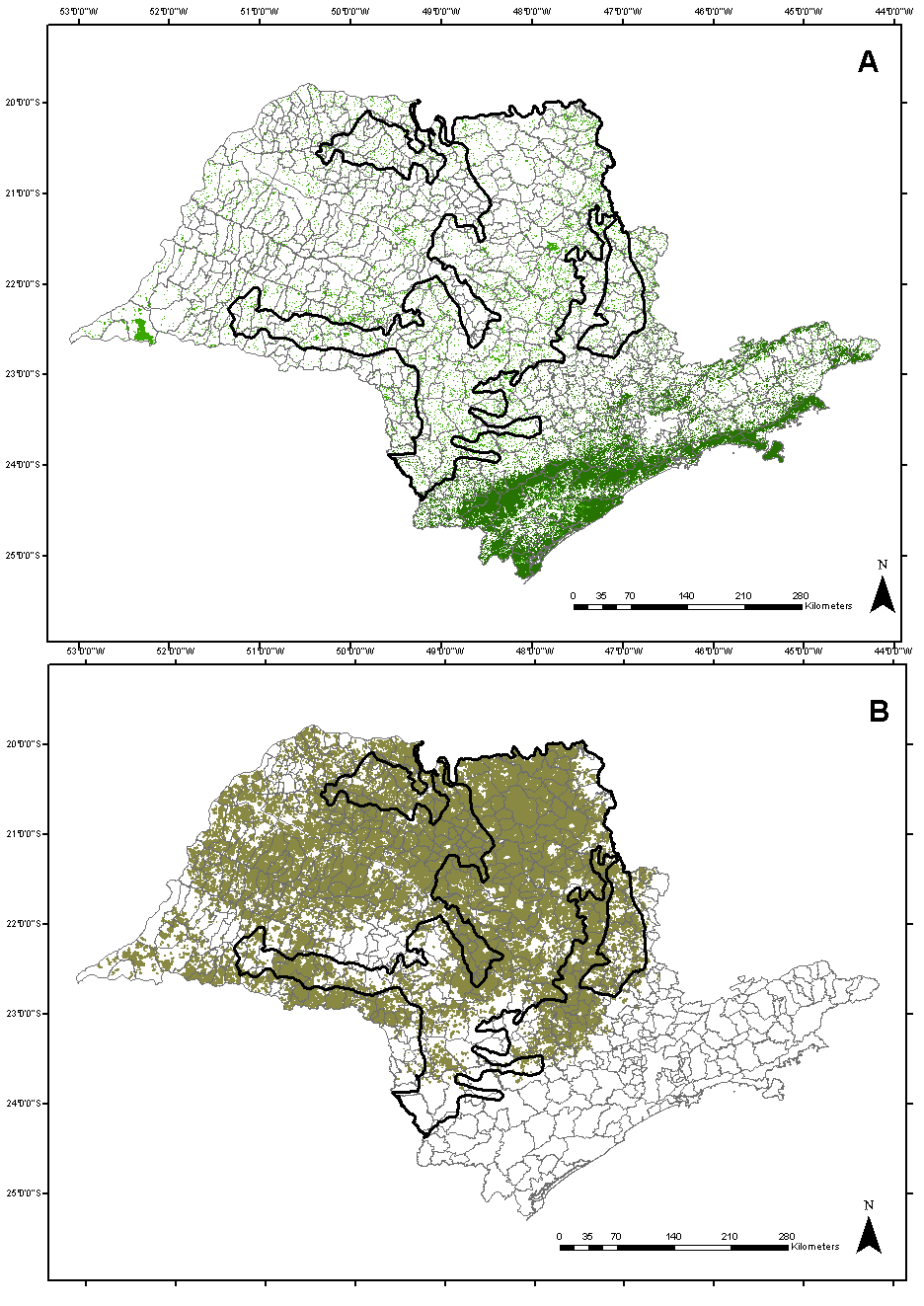


Figure S1. Amount of native vegetation (A) present in the state of São Paulo according to the Forestry Institute map of 2010, and (B) sugar cane plantation in 2011 according to the CaneSat/INPE 2011 (available at http://www.dsr.inpe.br/laf/Canesat/). Black line delineates the cerrado region.
