# Supplementary material for: Landscape, Environmental and Social Predictors of Hantavirus Risk in São Paulo, Brazil
Source: PLoS One. 2016 Oct 25;11(10):e0163459. doi: 10.1371/journal.pone.0163459 (PMC5079598; doi:10.1371/journal.pone.0163459)
Supplement: S2 Fig — Spatial representation of the minimum (A) and maximum (B) probability of Hantavirus infection risk for São Paulo State. (DOCX) [file pone.0163459.s009.docx]

Landscape, environmental and social predictors of Hantavirus risk in São Paulo, Brazil

Paula Ribeiro Prist^1*^, Maria Uriarte^2^, Leandro Reverberi Tambosi^1,2^, Amanda Prado^1^, Renata Pardini^3^, Paulo Sérgio D´Andrea^4^, Jean Paul Metzger^1^

**Supplementary** **Material**


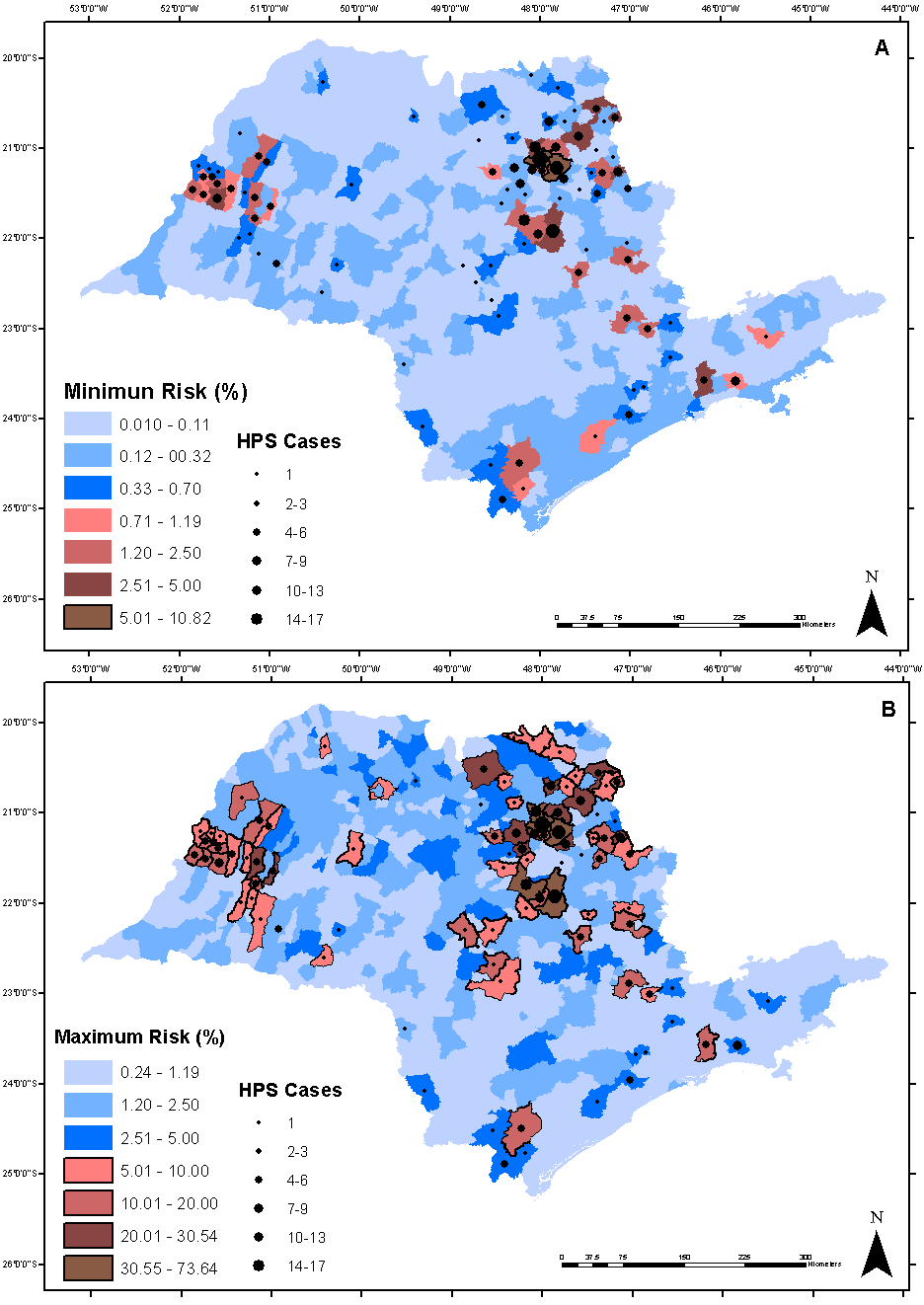


Figure S2. Spatial representation of the minimum (A) and maximum (B) probability of Hantavirus infection risk for São Paulo State and the number of HPS reported cases from 1993 to 2012. The municipalities with no symbol means municipalities with no reported cases of HPS. Black outlines indicate municipalities with medium to high risk (> 5%) of Hantavirus infection and where preventive effort should be allocated.
